# CT Pulmonary Angiography at Reduced Radiation Exposure and Contrast Material Volume Using Iterative Model Reconstruction and iDose^4^ Technique in Comparison to FBP

**DOI:** 10.1371/journal.pone.0162429

**Published:** 2016-09-09

**Authors:** Azien Laqmani, Maximillian Kurfürst, Sebastian Butscheidt, Susanne Sehner, Jakob Schmidt-Holtz, Cyrus Behzadi, Hans Dieter Nagel, Gerhard Adam, Marc Regier

**Affiliations:** 1 Department for Interventional and Diagnostic Radiology and Nuclear Medicine, University Medical Center Hamburg-Eppendorf, Hamburg, Germany; 2 Department of Medical Biometry and Epidemiology, University Medical Center Hamburg-Eppendorf, Hamburg, Germany; 3 Science & Technology for Radiology, Buchholz, Germany; Northwestern University Feinberg School of Medicine, UNITED STATES

## Abstract

**Purpose:**

To assess image quality of CT pulmonary angiography (CTPA) at reduced radiation exposure (RD-CTPA) and contrast medium (CM) volume using two different iterative reconstruction (IR) algorithms (iDose^4^ and iterative model reconstruction (IMR)) in comparison to filtered back projection (FBP).

**Materials and Methods:**

52 patients (body weight < 100 kg, mean BMI: 23.9) with suspected pulmonary embolism (PE) underwent RD-CTPA (tube voltage: 80 kV; mean CTDIvol: 1.9 mGy) using 40 ml CM. Data were reconstructed using FBP and two different IR algorithms (iDose^4^ and IMR). Subjective and objective image quality and conspicuity of PE were assessed in central, segmental, and subsegmental arteries.

**Results:**

Noise reduction of 55% was achieved with iDose^4^ and of 85% with IMR compared to FBP. Contrast-to-noise ratio significantly increased with iDose^4^ and IMR compared to FBP (p<0.05). Subjective image quality was rated significantly higher at IMR reconstructions in comparison to iDose^4^ and FBP. Conspicuity of central and segmental PE significantly improved with the use of IMR. In subsegmental arteries, iDose^4^ was superior to IMR.

**Conclusions:**

CTPA at reduced radiation exposure and contrast medium volume is feasible with the use of IMR, which provides improved image quality and conspicuity of pulmonary embolism in central and segmental arteries.

## Introduction

With advances in CT technology offering high temporal and spatial resolution, the diagnostic accuracy of CT pulmonary angiography (CTPA) has increased with reported sensitivities and specificities of 83–100% and 89–97%, respectively [1–3]. However, the utilization of CTPA increased dramatically, raising concerns with respect to radiation exposure [4–6]. As the high diagnostic value of CTPA has been outlined in numerous studies, the focus has been shifted to the optimization of CTPA protocols in order to comply to “as low as reasonably achievable” (ALARA) principles. Moreover, the use of iodinated contrast medium (CM) for CTPA examinations also remains a concern since it may contribute to contrast-induced nephropathy (CIN), especially in patients with pre-existing renal impairment [7, 8]. The risk of developing CIN is increased with higher volumes of iodinated CM [8]. Therefore an optimized CTPA protocol with a combination of both reduced radiation dose and reduced CM volume would be desirable. Low tube voltage CTPA protocols have shown the ability to reduce radiation dose and, by shifting the average x-ray photon energy closer to the k-absorption edge of iodine, to reduce CM volume in non-obese patients [9–12]. A drawback of low tube voltage is the increased image noise, which can impair image quality [13]. The increased image noise can be compensated by iterative reconstruction (IR). Previous studies have shown the feasibility of low KV CTPA examinations combined with IR, facilitating good image quality [14]. Recently, iterative model reconstruction (IMR) has been introduced, which offers a higher image noise reduction strength, thus a greater increase in CNR can be achieved, which can be potentially advantageous for reduction of CM volume. A combination of both, low kilo voltage CTPA protocol and IR may facilitate the reduction of both, radiation dose and CM volume, while maintaining image quality. Therefore, the aim of this study was to assess image quality of CTPA at reduced radiation exposure (RD-CTPA) and CM volume using the two different IR algorithms iDose^4^ and IMR in comparison to filtered back projection (FBP).

## Materials and Methods

### Patient population

Between November 2014 and January 2015, a total of 52 patients with a body weight less than 100 kg who were referred to our emergency department for a clinically indicated CTPA for suspected PE were included. Demographic patient data including sex, age, height, and weight were noticed. Body-Mass-Index (BMI) was calculated using the formula (patient weight (kg)/[patient height (m)]^2^).

The retrospective study was approved by the local Clinical Institutional Review Board (Ethic committee of the medical chamber of Hamburg) with a waiver of informed consent.

### CT pulmonary angiography acquisition protocol

All CTPA examinations were performed on a 256-slice MSCT scanner (Brilliance iCT, Philips, Best, The Netherlands) using the following parameters: tube voltage: 80 kV; detector collimation: 128 x 0.625 mm; pitch 0.915; tube rotation time: 0.4 s. The automatic exposure control system (automatic current selection (ACS)) combined with z-axis dose-modulation (Z-DOM)) were used with a quality reference tube charge setting of 130 effective mAs, resulting in a CTDI_vol_ setting of 2.6 mGy (without considering the additional dose saving effect of Z-DOM).

The CTPA scan was performed in a craniocaudal direction from the top of the aortic arch to the level of the bottom of the heart within a single breath hold at mid inspiration in order to avoid valsalva maneuvers. In cases of additionally suspected pneumonia, the scan area was extended.

The injection protocol consisted of intravenous administration of 40 mL contrast medium with an iodine concentration of 300 mg/ml (Imeron 300, Bracco Altana Pharma, Milan, Italy) at a flow rate of 4 mL/sec followed by a 20-ml saline flush at the same flow rate via a commercially available injector (Medrad®, Stellant® D, Bayer HealthCare, USA). The CT acquisition was triggered using a bolus tracking technique (Bolus Pro Ultra, Philips Healthcare) with the region of interest (ROI) placed in the pulmonary trunk. Data acquisition started with a delay of 4 seconds after exceeding a threshold level of 130 Hounsfield units (HU).

### iDose^4^ and IMR technique

iDose^4^ (Philips Healthcare, Cleveland, OH, US) represents a hybrid IR algorithm with 2 denoising components: an iterative maximum likelihood-type sinogram restoration based on Poisson noise distribution and a local structure model fitting on image data that iteratively decreases the noise [15, 16]. The iDose^4^ levels (1–7) define the increasing strength of noise reduction [14].

IMR is the latest IR approach introduced by Philips Healthcare and is an advanced, model and knowledge based IR technique that states to overcome the trade-off between image noise and spatial resolution that is typical for traditional FBP [17]. Noise reduction by up to factor 10 (17), which is even larger compared to other hybrid algorithms (factor up to 3 (10, 11, 13, 21)), provides virtually noise free images. But IMR aims at accounting for not only the noise behavior of the image but also the data statistics, image statistics, and system models during its iterative cycle. Hence, different aspects of image quality can be targeted, such as image smoothness, spatial resolution and artifacts. The vendor offers different “IMR definitions” (i.e. filter settings) for reconstruction of the images, whereby “BodyRoutine” (BR) is the recommended setting for CT angiography examinations. The IMR levels (1–3) define the increasing strength of noise reduction [16–18].

### Image reconstruction

All raw data were reconstructed with FBP, the HIR iDose^4^ and with a prototype implementation of the new knowledge-based IR algorithm IMR (Philips Healthcare, Cleveland, OH, US). Axial source images were reconstructed with a slice thickness of 1 mm and an increment of 0.5 mm using a standard body filter (B) for FBP and iDose^4^. For IMR, the recommended setting “BodyRoutine” (BR) was used. In order to compare different reconstruction levels of the two IR algorithms, two increasing iDose^4^ levels 4 (iDose^4^ L4) and 6 (iDose^4^ L6) and three increasing IMR levels 1 (IMR-BR1), 2 (IMR-BR2), and 3 (IMR-BR3) were used. The choice of iDose^4^ levels applied was based on a previous investigation [14]. Thus, six image series were reconstructed for each of the 52 patients, resulting in an overall number of 312 data sets.

### Quantitative image analysis

As described in detail previously [14], CT numbers (CT-N) of pulmonary arteries (PA), defined as the attenuation in Hounsfield units (HU), was measured in axial sections by placing circular regions of interest (ROIs) at nine different levels: the pulmonary trunk, right pulmonary artery, left pulmonary artery, right upper lobe artery, right middle lobe artery, right lower lobe artery, left upper lobe artery, left lingular artery and the left lower lobe artery, respectively. To minimize any bias from a single measurement, each selected artery was measured at three adjacent 1-mm slices, and the results were averaged for further calculations. Based on the CT-N of the nine different levels, an average pulmonary vessel CT-N (CT-N_vessel_) was calculated.

Objective image noise (OIN) was defined as the mean of the standard deviations (SD) of CT-N. For measuring mean background CT-N (CT-N_background_), circular ROIs were placed within the pectoral muscles on both sides, and the attenuation was averaged. Contrast-to-noise ratio (CNR) was calculated by use of the following formula: CNR = (CT-N vessel−CT-N _background_)/ OIN. To ensure measurement accuracy, the initial ROI was placed on the image reconstructed with FBP and was then copied and pasted onto the corresponding five data sets of iDose^4^ and IMR reconstructions.

### Qualitative image analysis

Two radiologists with 7 and 11 years of experience in CTPA, who were blinded to the reconstruction technique applied, independently evaluated each data set by using axial sections and standard CTPA window settings (window width, 450 HU; window center, 50 HU). To standardize subjective evaluation, images obtained from five patients not included in the study were read prior to assessment in consensus. For image analysis, all 312 reconstructed image series were randomized and rendered anonymous. The readers were allowed to zoom in and change the window level and width for assessing structures ad libitum.

All RD-CTPA reconstructions were rated according to a 5-point scale (1 indicating worst through to 5 indicating best) for subjective image quality, subjective image noise, and blotchy image appearance using evaluation criteria being published previously [14]. The obtained five-point scoring system is described in Table 1.

**Table 1:** Five-point Scoring System of Different Image Quality Characteristics for Observer Study.

Score	Subjective image quality	Subjective image noise	Blotchy image appearance
**5**	excellent image quality, no artifacts, full diagnostic confidence	no perceived noise	no blotchy appearance
**4**	good image quality, minor artifacts, not affecting diagnostic confidence	minor noise	minor blotchy appearance
**3**	moderate image quality, increased artifacts, impairment of diagnostic confidence	moderate noise	moderate blotchy appearance
**2**	reduced image quality, substantial artifacts, limited diagnostic confidence	extensive noise	increased blotchy appearance
**1**	poor image quality, major artifacts, no diagnosis possible	major noise	major blotchy appearance

### Conspicuity of pulmonary embolism

Assessment of conspicuity of PE followed the method previously described by Laqmani et al. [14] using a three-point scale as follows: 1, subtle, may be an artifact; 2, sufficient, filling defect definable; and 3, excellent, filling defect clearly definable. The anatomic location of any filling defects and the number of the affected PA were recorded. The location of PE was classified as either central/lobar (pulmonary trunk, right/ left PA and lobar PA), segmental or subsegmental. To identify PA, the nomenclature outlined by Remy-Jardin et al [19] was used, which was adapted by Ghaye et al [20].

Subsequently, a consensus readout session was performed for all data sets in a side-by-side comparison in order to ensure the assessment of all filling defects. These results were used as the reference standard.

### Radiation dose estimation

The volume weighted CT dose index (CTDI_vol_), the effective tube charge and the dose-length product (DLP) of each examination were recorded as provided by the scanner. The estimates for the calculation of the effective dose (E) were based on the European Guidelines on quality criteria in CT using the formula E = E_*DLP*_ * DLP, where E is the effective dose in mSv and E_*DLP*_ = 0.014 mSv / mGy*cm (dose length coefficient for chest) [21].

### Statistical analysis

Sample characteristics are given as absolute and relative frequencies or mean +/- standard deviation, whichever is appropriate. To account for the repeated measurement structure of the data, defined by three 1 mm adjacent image slices and nine different vessels per patient and reconstruction level, multilevel mixed-effects linear regression was calculated for the quantitative parameters. In this model, the reconstruction level was included as predictor for the quantitative parameters.

For the qualitative Likert scale an analogous model was used while the repeated measurements were defined by the ratings of the two readers. To evaluate the subjective image quality, the interaction between reconstruction level and each subjective image quality characteristic was included as predictor. For the evaluation of conspicuity of PE, the interaction between reconstruction level and the three different vessel groups was included as predictor. All models were controlled adjusted for BMI, age, gender, tube charge and effective dose. The results were estimated as marginal means, which are represented in graphs with 95% confidence intervals (95%-CIs).

The quantitative parameter CT-N_vessel_ as well as the qualitative scores for filling defect conspicuity were analysed in an analogous manner with the interaction between vessel, respective vessel groups (central/lobar, segmental, and subsegmental), and reconstruction technique and level as fixed effect. If this interaction was insignificant, only the main effects were included. Posthoc tests for comparison of marginal means were calculated with contrast tests, using Wald tests with correction for multiplicity by Bonferroni. P-values <0.05, two sided were considered significant. All analyses were computed using Stata 14.1 (STATA Corporation, College Station, Texas, USA).

## Results

### Population characteristics

The study population consisted of 23 women and 29 men. The mean age of the 52 patients was 65.3 ± 17.5 years (range: 20 to 94), the mean body weight and height were 71.7 ± 11.3 kg (range: 49 to 95 kg) and 173 ± 7.5 cm (range: 155 to 188 cm), respectively. The calculated mean BMI was 23.9 ± 3 kg/m^2^ (range: 16.9 to 30.4).

### Radiation dose

The mean exposure was 97 ± 33 effective mAs (range: 45 to 191), the mean CTDI_vol_ and DLP were 1.9 ± 0.6 mGy (range: 0.9 to 3.7) and 66 ± 27 mGy*cm (range: 22 to 145), respectively. These resulted in a mean effective dose of 0.9 ± 0.4 mSv (range: 0.3 to 2.0). The average net (i.e. imaged) scan length was 24.9 ± 6.1 cm (range: 15.1 to 35.9).

### Quantitative results

**Fig 1:**
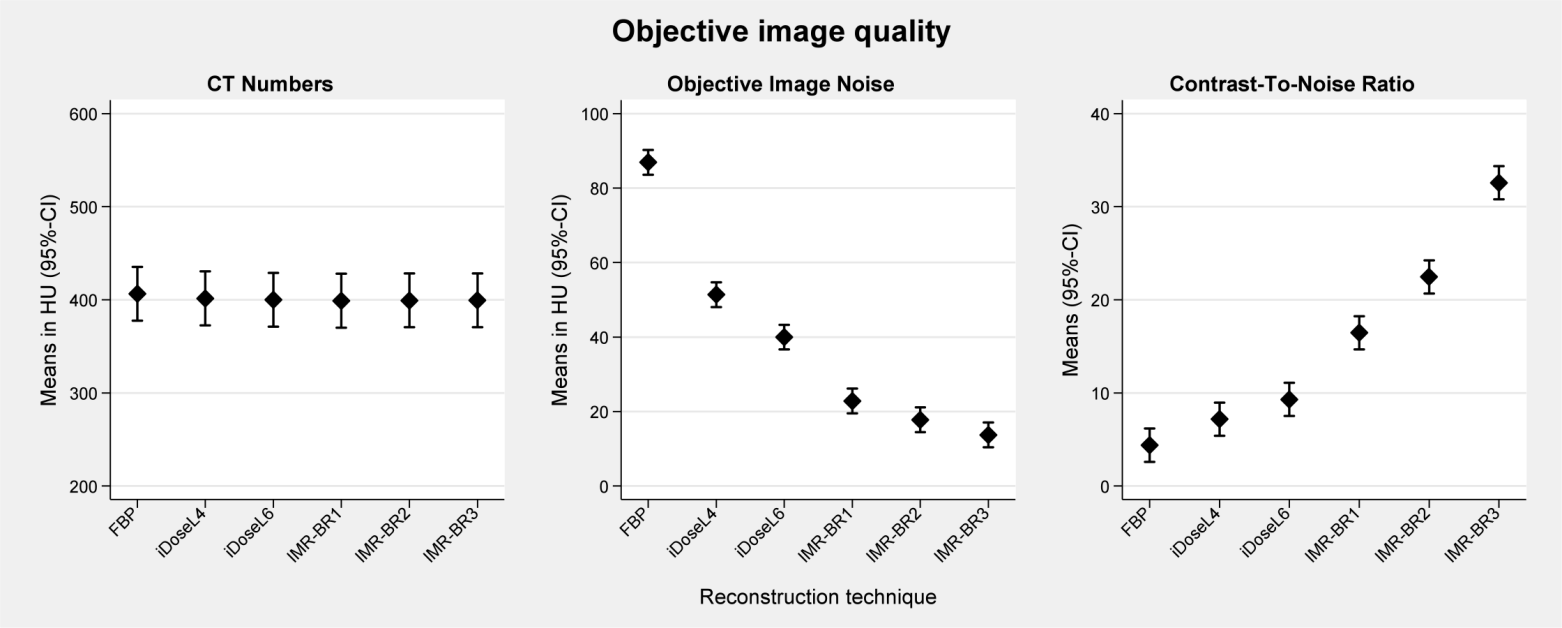
Quantitative analysis of RD-CTPA being reconstructed with FBP, iDose^4^ and IMR. Error bars represent the 95% confidence interval.

Quantitative results (±95%-CI) are graphically displayed in Fig 1. None of the confounder showed a significant effect on the quantitative parameters. The mean CT-N_vessel_ in RD-CTPA studies reconstructed with FBP was slightly but not significantly higher than that in reconstructions with iDose^4^ and IMR (CT-N_vessel_: FBP: 406.5 HU; iDoseL4 401.5 HU; iDoseL6: 400 HU; IMR-BR1: 399 HU; IMR-BR2: 399.3 HU; IMR-BR3: 399.4).

**Fig 2:**
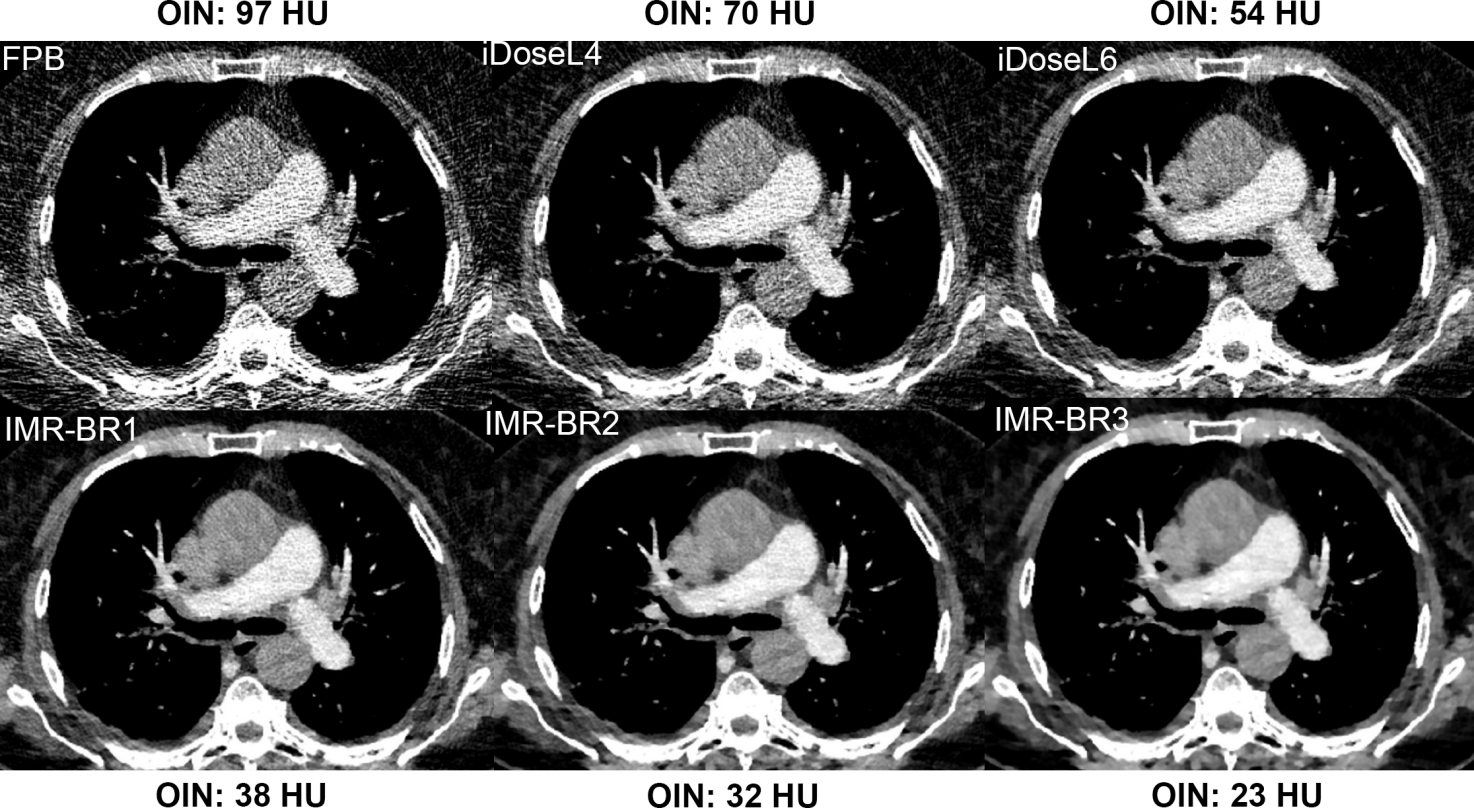
Objective image noise reduction with application of iDose^4^ and IMR compared with FBP. Objective image noise of this RD-CTPA significantly decreased from FBP (97 HU) to iDoseL4 (70 HU), to iDoseL6 (54 HU), to IMR-BR1 (38 HU), to IMR-BR2 (32) and IMR-BR3 (23 HU).

OIN was reduced up to 41% to 55% with iDose^4^ and up to 74% to 85% with IMR compared to FBP (p<0.001) (Figs 1 and 2). CNR calculations showed a statistically significant progressive increase between FBP, the different iDose^4^ and IMR reconstructions. FBP had the lowest CNR value with 4.4 and IMR-BR3 showed the highest CNR value with 32.6 (p < 0.001).

### Qualitative results

**Fig 3:**
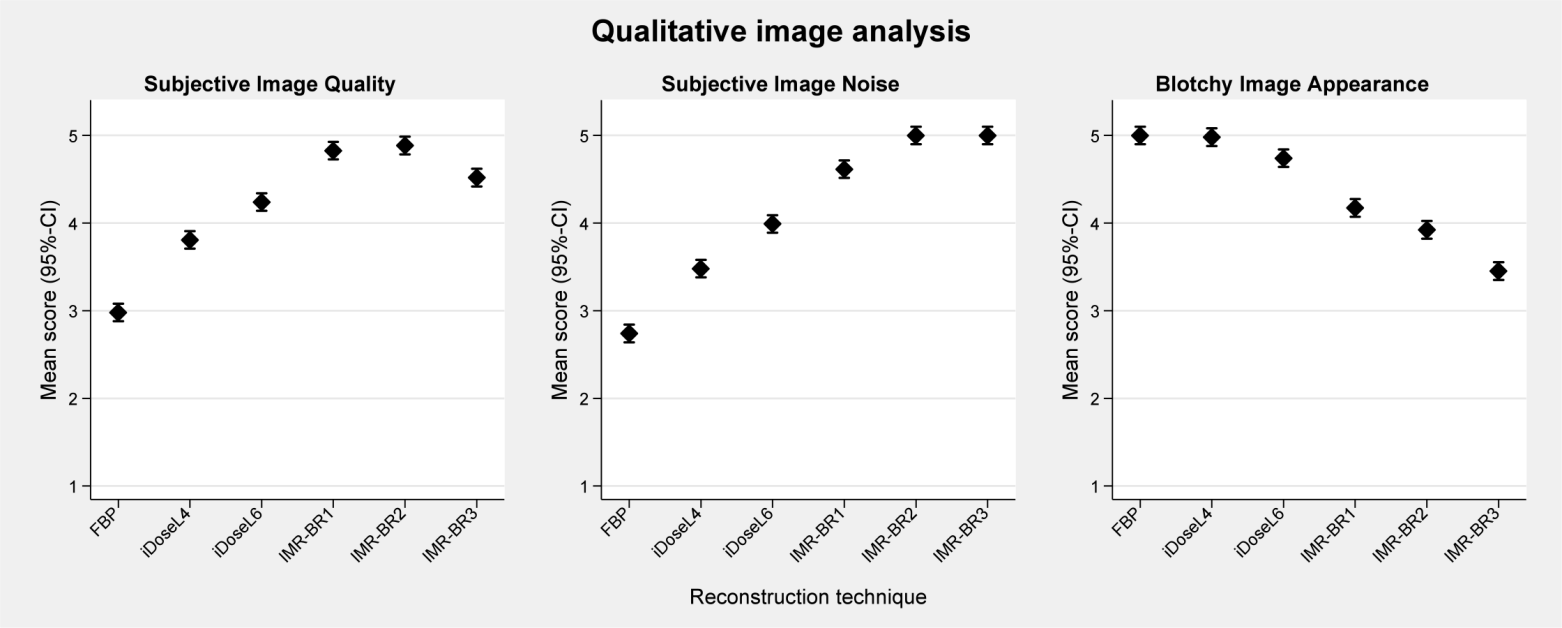
Qualitative analysis of RD-CTPA being reconstructed with FBP, iDose^4^ and IMR. The plots show the mean scores for subjective image quality, subjective image noise and blotchy image appearance. Error bars represent the 95% confidence interval. Subjective 5-point grading scale (1 indicating worst through 5 indicating best).

Mean scores (95%-CIs) of qualitative image analysis are graphically displayed in Fig 3. None of the confounder showed a significant interaction with reconstruction technique/level or image quality.

**Fig 4:**
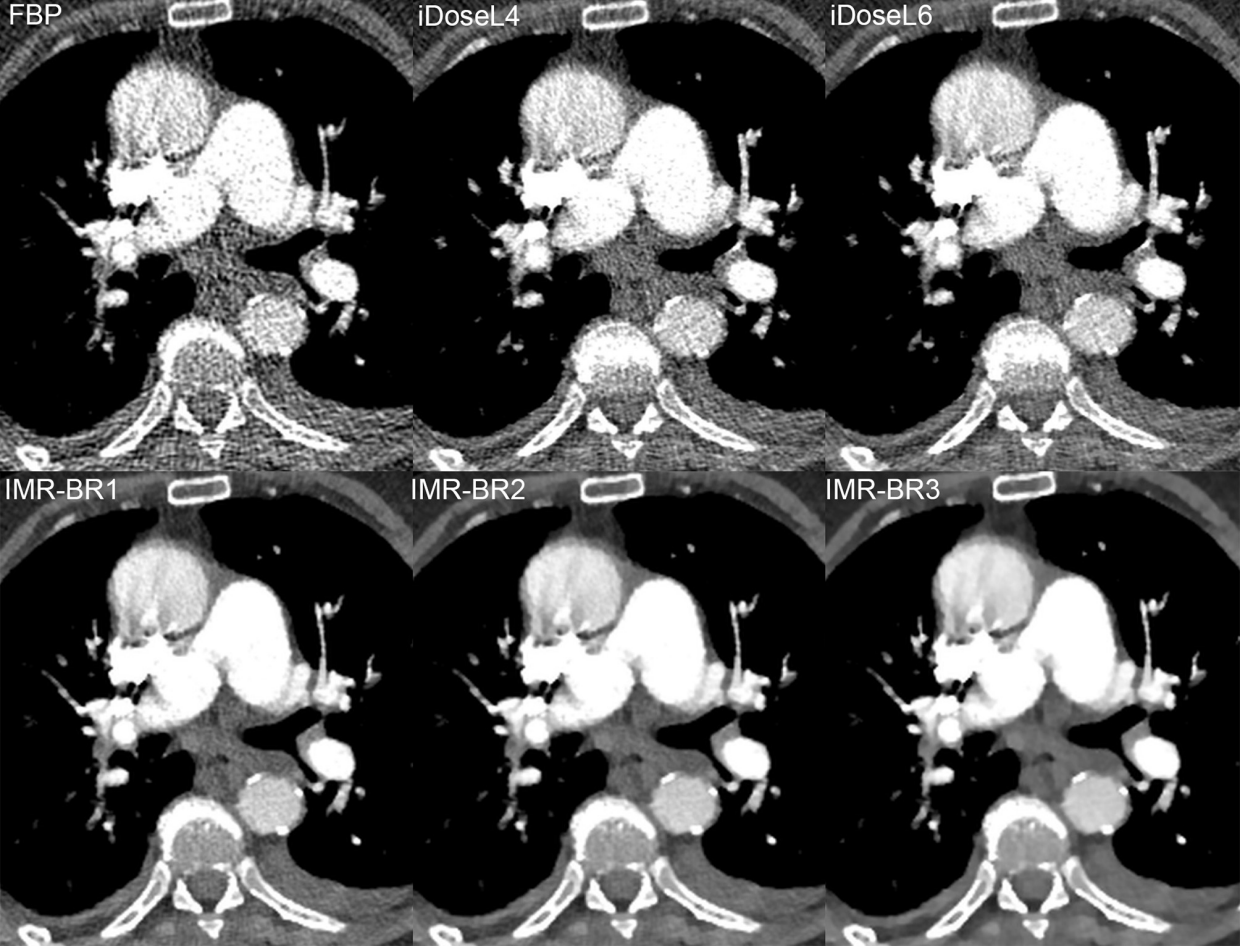
Image quality, image noise and image appearance of RD-CTPA being reconstructed with FBP, iDose^4^ and IMR. Transverse RD-CTPA image reconstructed with FBP, iDoseL4, iDoseL6, IMR-BR1, IMR-BR2 and IMR-BR3. Subjective image quality and image noise improved with the application of iDose^4^ and IMR compared with FBP. Simultaneously, blotchy appearance increases moderately with application of iterative reconstruction.

Subjective image quality improved significantly with the application of iDose^4^ and IMR compared with FBP (p<0.001) (Figs 3 and 4). While CTPA images reconstructed with FBP (mean score 2.9; [2.8–3.1]) were graded as having the lowest image quality, the IMR level 1 (mean score 4.8; [4.7–4.9]) and the IMR level 2 (mean score: 4.9; [4.8–5]) were rated as providing the best image quality with no significant differences between these two IMR levels.

The perception of subjective image noise (Figs 3 and 4) significantly improved with application of IMR in comparison with FBP and to a lower level also in comparison with iDose^4^ (p<0.001). The middle and the highest IMR levels (IMR-BR1 and IMR-BR2) were graded to have no perceived image noise. A minor blotchy image appearance was noticed with the use of low and middle IMR levels, which did not affect diagnostic confidence. This blotchy smoothed image appearance was further enhanced with application of the highest IMR level 3 (p< 0.001), mildly limiting diagnostic confidence of the readers (mean score: 3.5; [3.4–3.6]) (Figs 3 and 4).

There was an excellent interobserver agreement for the evaluation of image quality between both readers (ICC: 0.001; reliability 99%).

### Pulmonary embolism conspicuity results

Ten patients with PE were identified, whereas filling defects were identified in 1 main pulmonary artery, 33 central/lobar PA (8 right and left pulmonary arteries, 25 lobar arteries), 61 segmental arteries and in 36 subsegmental arteries. The final side-by-side readout of corresponding FBP, iDose^4^ and IMR data sets did not disclose any additional filling defects in any of the different reconstructions.

**Fig 5:**
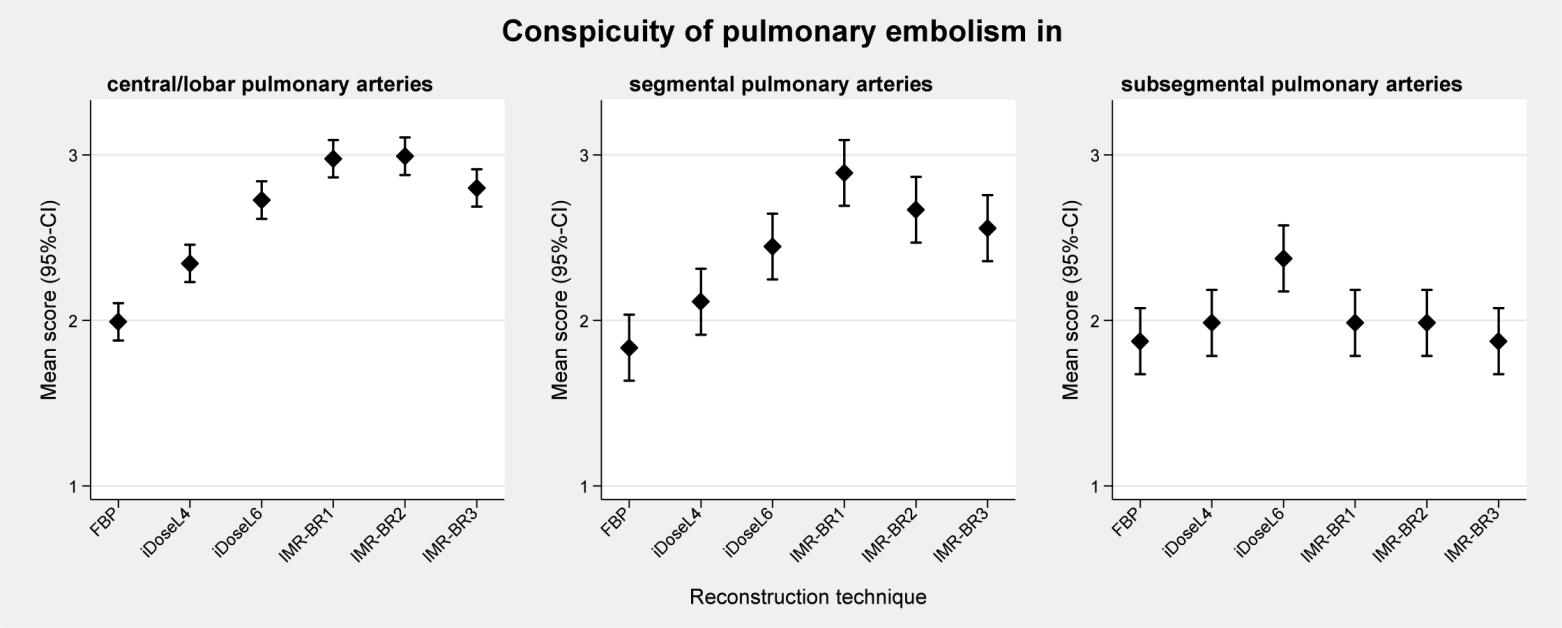
Qualitative analysis of conspicuity of pulmonary embolism in central/lobar, segmental and subsegmental pulmonary arteries. The plots show the mean subjective image scores (error bars represent the 95% confidence interval). Subjective 3-point grading scale: 1, subtle, may be an artifact; 2, sufficient, filling defect definable; and 3, excellent, filling defect clearly definable.

**Fig 6:**
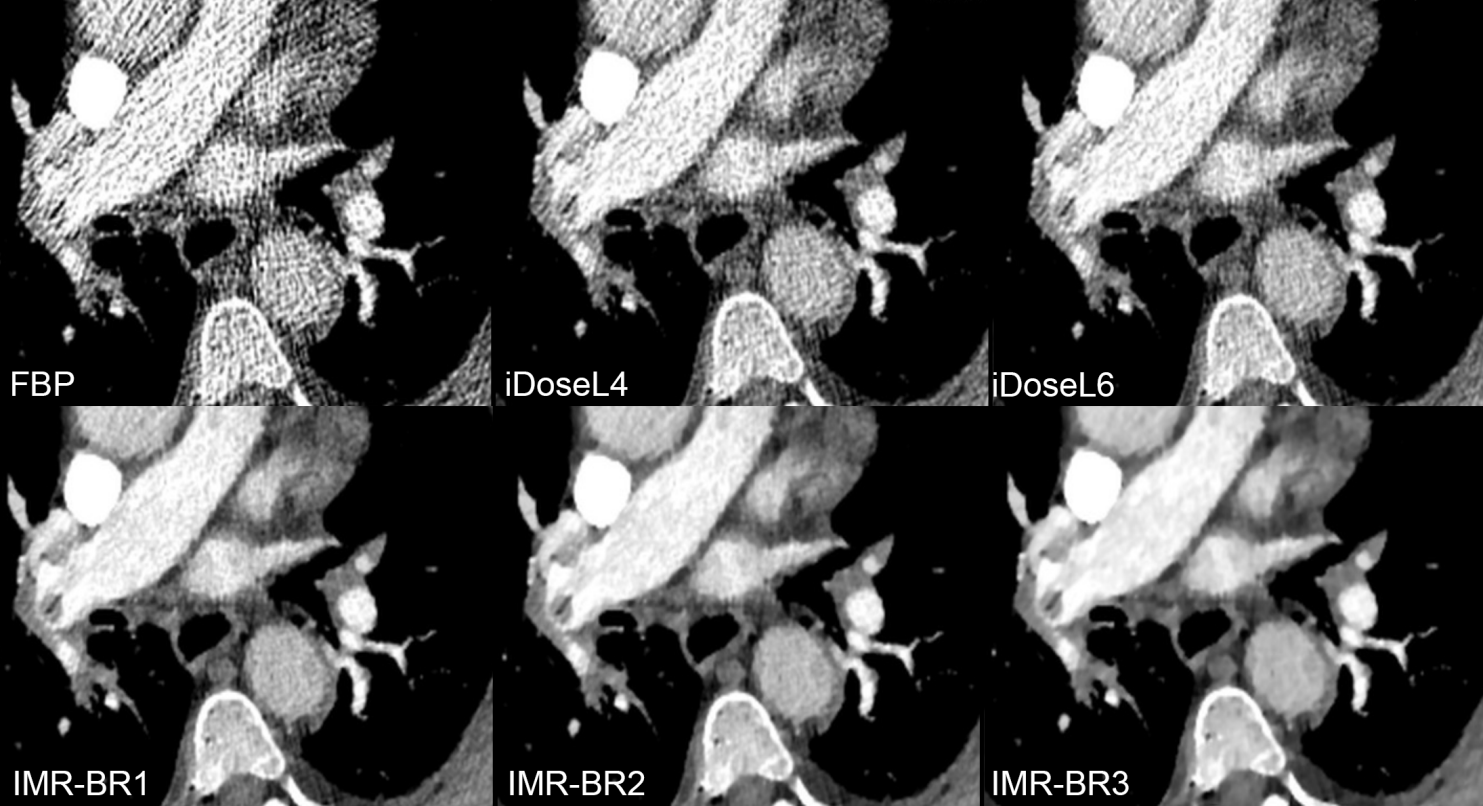
Conspicuity of lobar pulmonary embolism in RD-CTPA being reconstructed FBP, iDose^4^ and IMR. Transverse RD-CTPA image reconstructed with FBP, iDoseL4, iDoseL6, IMR-BR1, IMR-BR2 and IMR -BR3 demonstrating a right-sided pulmonary embolism with filling-defect in the right lower lobe artery being obscured at FBP. With application of the iterative reconstruction algorithms iDose^4^ and IMR a significant decrease of image noise and streak artifacts was achieved, enabling a better conspicuity of the filling defect with IMR and to lower extent also with iDose^4^ compared to FBP.

**Fig 7:**
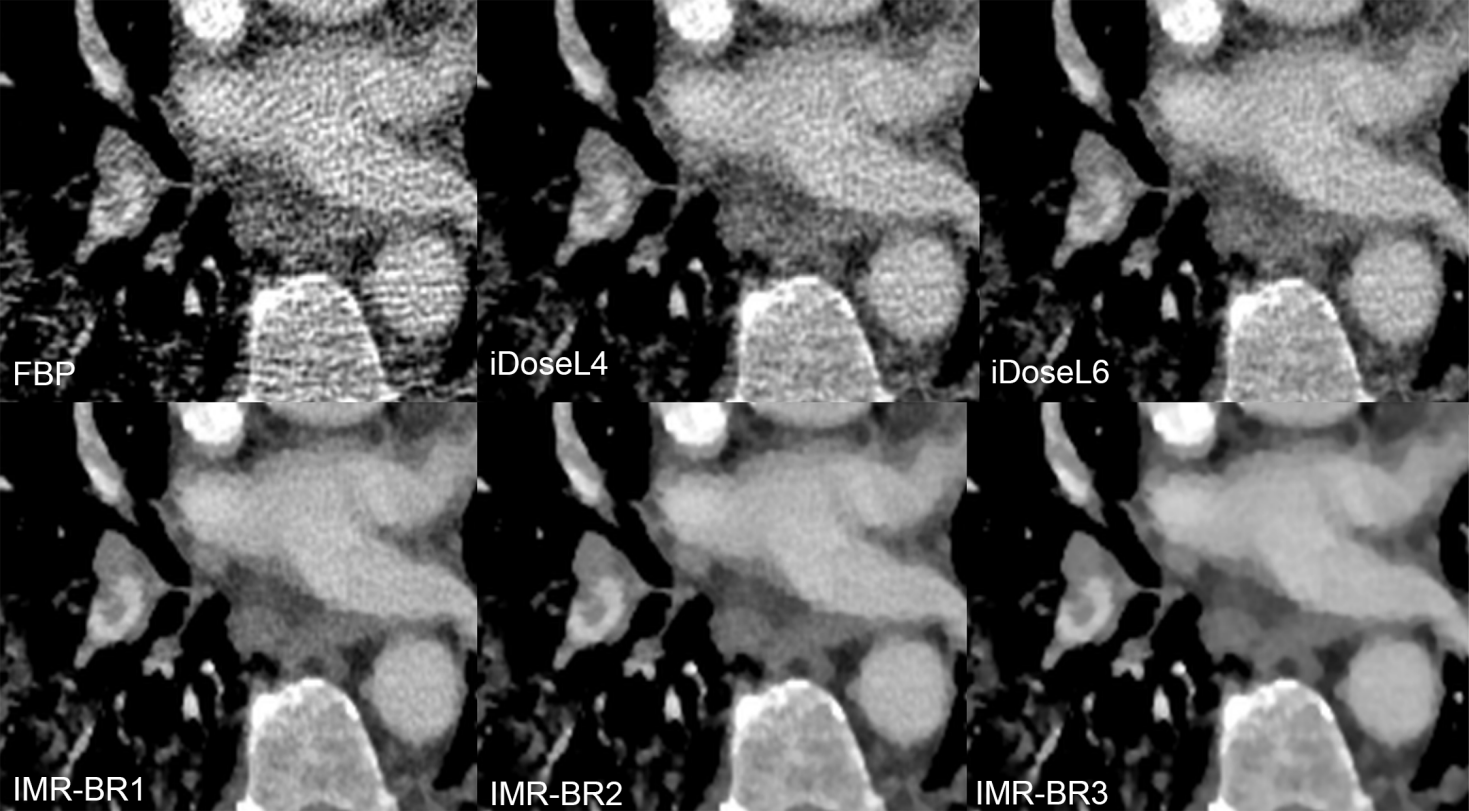
Conspicuity of segmental pulmonary embolism in RD-CTPA being reconstructed FBP, iDose^4^ and IMR. Transverse RD-CTPA image reconstructed with FBP, iDoseL4, iDoseL6, IMR-BR1, IMR-BR2 and IMR -BR3 demonstrating a right-sided segmental pulmonary embolism. Conspicuity of the filling defect improved with application of the iterative reconstruction algorithms iDose^4^ and IMR.

Mean scores (95%-CIs) of qualitative results of conspicuity of PE are graphically displayed in Fig 5. In central/lobar (Fig 6) and segmental PA (Fig 7), the conspicuity of PE improved with use of IMR compared with FBP and to a lower level also compared with iDose^4^ (p< 0.05). IMR-BR1 was rated to be the best reconstruction setting for PE conspicuity in central/lobar and also in segmental PA.

**Fig 8:**
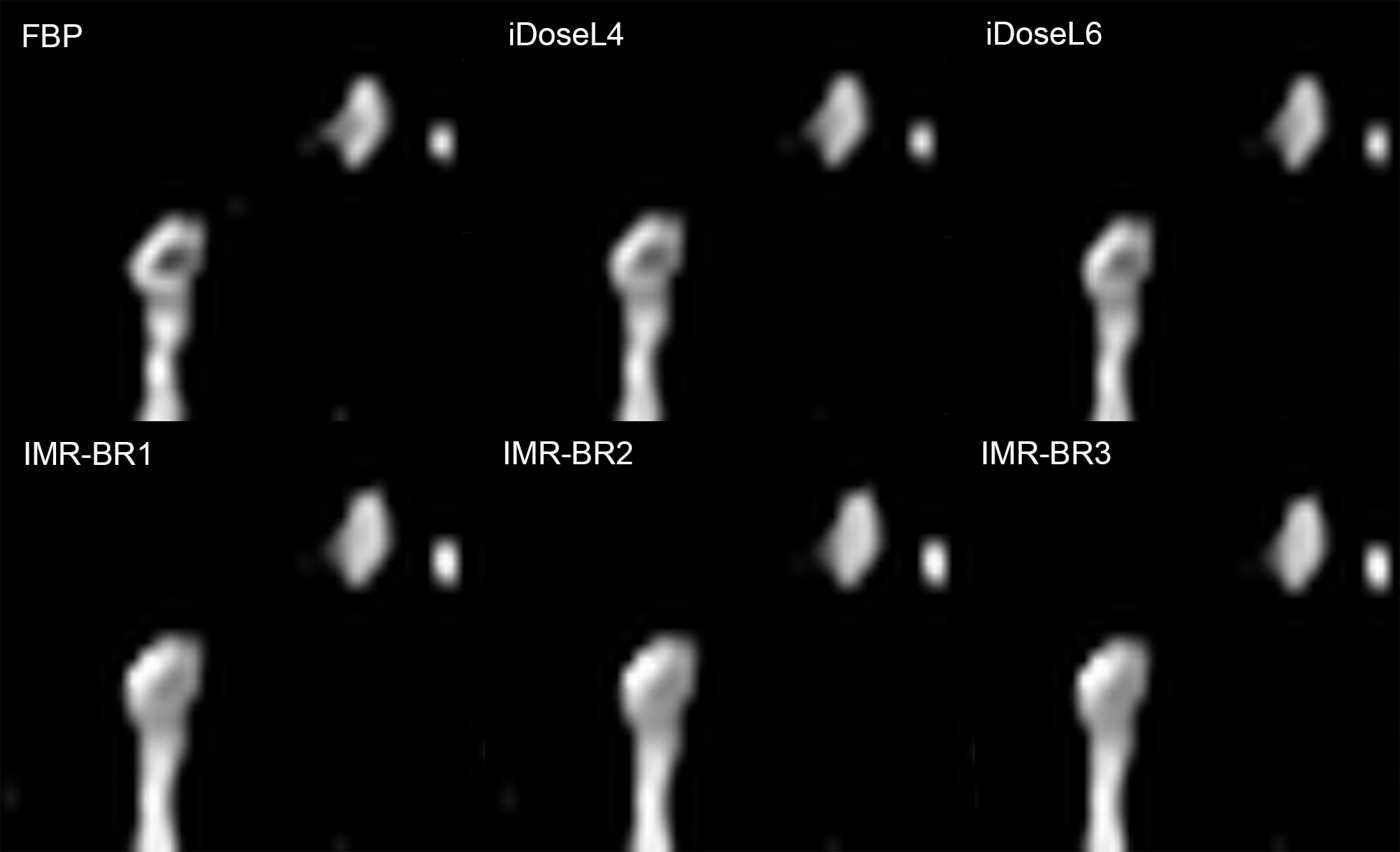
Conspicuity of subsegmental pulmonary embolism in RD-CTPA being reconstructed FBP, iDose^4^ and IMR. Detailed enlargement of a subsegmental pulmonary artery with embolus. RD-CTPA image reconstructed with FBP, iDoseL4, iDoseL6, IMR-BR1, IMR-BR2 and IMR -BR3. Whereas the filling defect is well definable in FBP images, it’s conspicuity slightly decreases with iDose^4^ and markedly decreases with IMR.

For the assessment of PE in subsegmental PA, iDose^4^ level L6 (mean score: 2.4; [2.2–2.6]) was rated to have the best image quality. iDoseL6 was superior to FBP (p< 0.05) and to IMR-BR3 (p: 0.004) (Fig 8).

There was an excellent interobserver agreement for the conspicuity of PE between both readers (ICC: 0.015; reliability 98.5%).

## Discussion

In this study we evaluated a dose-saving protocol with 80 kVp as well as a reduced amount of CM in combination with IR. Our results showed that such a combined RD-CTPA protocol and reduced CM amount provide good to excellent image quality in non-obese patients by the additional application of IR algorithms. The knowledge-based IR algorithm IMR yielded the best objective and subjective image quality for the assessment of pulmonary arteries and for the depiction of central, lobar and segmental emboli at a reduced effective radiation dose of 0.92 ± 0.3 mSv.

Optimized radiation dose and contrast medium CTPA protocols are of significant importance, since the use of CTPA is steadily increasing within the last years [4, 22]. A study from one large academic institution reported a fivefold increase in CTPA examinations from 2001 to 2007 [4]. A multi-centric study estimated that one in 330 females undergoing CTPA at the age of twenty years will develop radiation-induced cancer [22]. Furthermore, the contrast medium administration required for CTPA bears the potential risk of CIN. Although the risk of CIN may be overestimated [23], it’s incidence and severity is directly related to the contrast dose [24]. The most suitable approach to reduce both the radiation dose and the CM volume during CT is to lower the tube voltage [25–27]. Thus, an increase in attenuation values of iodinated CM and CNR can be achieved, which can facilitate both the improvement of image quality and the reduction of CM volume [27]. Despite the increase of CNR due to increase in attenuation values, the simultaneously increase in image noise is one disadvantage of low tube voltage and can limit a further dose reduction [14, 28]. Previous studies have reported high image noise values for 80 kVp CTPA protocols [11, 12] if the effective dose was reduced [12]. To address image noise, recent studies have combined low kilo voltage with IR in CTPA protocols. Lu et al. [10] reported sufficient image quality in a high-pitch 80 kVp CTPA protocol with an effective radiation dose of 0.9 ± 0.2 mSv by application of the sinogram affirmed iterative reconstruction algorithm. Pontana et al. [29] demonstrated that raw data-based iterative reconstruction provided improved OIN levels in low-voltage half-dose CT angiograms when compared with standard-dose FBP images with a reduced effective radiation dose of 1.03 mSv. Our results demonstrate that a reduced radiation dose to less than 1 mSv can be achieved using an 80 kVp RD-CTPA protocol and that by application of IMR both objective and subjective image quality can be effectively improved at this reduced dose setting. Quantitative measurements demonstrated a substantial noise reduction capability of IMR, which was even superior to the hybrid IR iDose^4^. IMR provided a noise reduction of up to 85% and 66% compared to FBP and to iDose^4^, respectively. CNR calculations demonstrated that IMR provided the highest values, which were significantly higher than FBP and iDose^4^. According to the literature, a minimum CNR of five is required for a reliable detection of PE [30]. In our study, FBP provided only a CNR of 4.1 whereas IMR provided a CNR of 26.8 [30]. This significant improvement in CNR is based on the IMR induced noise reduction. Two recent studies on low kilo voltage coronary CT angiography using IMR supported our results by reporting significant noise reductions and improvement of CNR and image quality in angiographic studies [31, 32]. IMR is a knowledge-based IR approach in which reconstruction becomes a process of optimization that takes into account the data statistics, image statistics, as well as system models [17]. It generates optimal images by iteratively minimizing the difference between acquired data and their ideal form, providing noise and artifact free images [17, 31]. The consecutive improvement of CNR offers the opportunity to reduce the amount of CM. Zhang et al. [32] reported the feasibility of CM volume reduction in combination with a low kilo voltage coronary CT angiography protocol by the use of IMR. The CM volume commonly applied in CTPA of non-obese patients varies between 60 and 100 ml [29, 33, 34]. According to the literature, a minimum attenuation value of 211 to 250 HU in PA permits reliable exclusion of acute and chronic thromboembolism [35, 36]. The mean attenuation of about 400 HU inside the PA in our study using only 40ml of CM markedly exceeded these values. Our results show that an excellent enhancement of the PA can be achieved using the low CM amount of 40 ml, thus reducing the risk of CIN. A recent study by Lu et al. [10] suggested that even a further reduction of CM amount could be achieved in high-pitch CTPA at 80 kVp when being combined with IR by offering sufficient image noise reduction. However, previous studies reported that excessive noise reduction could result in an over-smoothing and blurring of the images [14, 37]. In our study, overall subjective image quality improved with application of both IR algorithms iDose^4^ and IMR compared to FBP, whereas the best overall image quality was rated at the low and middle IMR strength levels. However, the high iDose^4^ level 6 provided a distinct blotchy appearance, which was further enhanced applying the highest IMR level 3. The use of low and middle IMR levels 1 and 2 resulted in a minor blotchy image appearance, which did not affect diagnostic confidence.

iDose^4^ can overcome the change in image appearance to some degree due to its hybrid iterative algorithm. A previous phantom study showed that iDose^4^ reduces the noise power spectrum uniformly over the entire spatial frequency ranges, thus providing an image appearance which is comparable to what radiologists have been used to over the past three decades [38]. Knowledge-based IR such as IMR is non-linear, and the spatial resolution can vary with the object contrast and noise level [32]. However, in a phantom study obtained by Mehta et al., IMR provided both improvement of low and high-contrast spatial resolution, respectively [17]. These experimental results are not fully consistent with our clinical study, as the conspicuity of small subsegmental emboli degraded with IMR compared to FBP and iDose^4^. The ability to distinguish between noise and structures obviously diminishes with increasing IR strength when structures and differences in density become small. This has already been reported in a number of other studies, assessing iterative reconstruction algorithms of various vendors [39–41]. As a consequence the edges of the filling defects become diffuse with IMR, making it difficult to resolve small structures as small subsegmental emboli, even though the overall image noise is remarkably decreased by IMR. This presumed decrease of structure edges with IMR did not compromise the conspicuity of filling defects in central/ lobar and segmental PA, rather the application of IMR resulted in higher conspicuity of filling defects in comparison to FBP and iDose^4^. Here, IMR benefits from its superior strength in noise and streak artifact reduction.

Streak artifacts caused by mediastinal structures, contrast within the cardiac chambers, inflow of highly concentrated CM into the brachiocephalic vein and superior vena cava during CTPA are a well-reported concern and can obscure the pathways of the central and segmental PA [42, 43]. These artifacts are markedly reduced by IMR, thus enabling a better conspicuity of the underlying structures such as filling defects in central and segmental PA.

Our study has several limitations. Firstly, the IMR prototype was not officially cleared for clinical use; hence it was not possible to reduce the dose beyond the level that is appropriate for iDose4. As a consequence we cannot state if a further radiation dose reduction would also provide sufficient image quality when being combined with IMR. Secondly, we limited patient enrollment to individuals with a maximum body weight of 100 kg. Hence, further investigations have to be obtained to evaluate the influence of IR on reduced dose settings in CTPA protocols in obese patients exceeding 100 kg. Thirdly, patients had not undergone the gold standard of lung scintigraphy. Thus, false negative findings cannot definitely be excluded. Fourthly, even though the subjective image evaluation was obtained in a blinded manner, complete blinding to the image reconstruction techniques was not feasible because of the inherent image texture differences among the three reconstruction methods.

In conclusion, CTPA at reduced radiation exposure and contrast medium volume is feasible with application of IMR, which provides improved image quality and conspicuity of pulmonary embolism in central and segmental arteries.
